# Anticipating Tomorrow: Tailoring Parkinson's Symptomatic Therapy Using Predictors of Outcome

**DOI:** 10.1002/mdc3.14089

**Published:** 2024-05-30

**Authors:** Ronald B. Postuma, Daniel Weintraub, Tanya Simuni, Mayela Rodríguez‐Violante, Albert F.G. Leentjens, Michele T. Hu, Alberto J. Espay, Roberto Erro, Kathy Dujardin, Nicolaas I. Bohnen, Daniela Berg, Tiago A. Mestre, Connie Marras

**Affiliations:** ^1^ Department of Neurology Montreal Neurological Institute, McGill University Montreal Quebec Canada; ^2^ Departments of Psychiatry and Neurology, Perelman School of Medicine at the University of Pennsylvania; Parkinson's Disease Research, Education and Clinical Center (PADRECC) Philadelphia Veterans Affairs Medical Center Philadelphia Pennsylvania USA; ^3^ Feinberg School of Medicine Northwestern University Chicago Illinois USA; ^4^ Movement Disorders Clinic National Institute of Neurology and Neurosurgery Mexico City Mexico; ^5^ Department of Psychiatry Maastricht University Medical Center Maastricht The Netherlands; ^6^ Nuffield Department of Clinical Neurosciences, Neurology Department Oxford University and John Radcliffe Hospital Oxford United Kingdom; ^7^ James J. and Joan A. Gardner Family Center for Parkinson's Disease and Movement Disorders, Department of Neurology University of Cincinnati Cincinnati Ohio USA; ^8^ Department of Medicine, Surgery and Dentistry “Scuola Medica Salernitana”, Neuroscience Section University of Salerno Baronissi Italy; ^9^ Neurology and Movement Disorders Department University of Lille, Inserm, Lille Neurosciences and Cognition, CHU‐Lille Lille France; ^10^ Departments of Radiology and Neurology University of Michigan, University of Michigan Udall Center, Ann Arbor VAMC Ann Arbor Michigan USA; ^11^ Department of Neurology Christian‐Albrechts‐University Kiel Germany; ^12^ Division of Neurology, Department of Medicine University of Ottawa, The University of Ottawa Brain and Research Institute Ottawa Ontario Canada; ^13^ Parkinson's Disease and Movement Disorders Clinic The Ottawa Hospital, The Ottawa Hospital Research Institute Ottawa Ontario Canada; ^14^ Edmond J. Safra Program in Parkinson's Disease and the Morton and Gloria Shulman Movement Disorders Clinic Toronto Western Hospital, University Health Network Toronto Ontario Canada

**Keywords:** Parkinson's disease, personalized medicine, prediction, subtypes, therapy

## Abstract

**Background:**

Although research into Parkinson's disease (PD) subtypes and outcome predictions has continued to advance, recommendations for using outcome prediction to guide current treatment decisions remain sparse.

**Objectives:**

To provide expert opinion‐based recommendations for individually tailored PD symptomatic treatment based on knowledge of risk prediction and subtypes.

**Methods:**

Using a modified Delphi approach, members of the Movement Disorders Society (MDS) Task Force on PD subtypes generated a series of general recommendations around the question: “Using what you know about genetic/biological/clinical subtypes (or any individual‐level predictors of outcome), what advice would you give for selecting symptomatic treatments for an individual patient now, based on what their subtype or individual characteristics predict about their future disease course?” After four iterations and revisions, those recommendations with over 75% endorsement were adopted.

**Results:**

A total of 19 recommendations were endorsed by a group of 13 panelists. The recommendations primarily centered around two themes: (1) incorporating future risk of cognitive impairment into current treatment plans; and (2) identifying future symptom clusters that might be forestalled with a single medication.

**Conclusions:**

These recommendations provide clinicians with a framework for integrating future outcomes into patient‐specific treatment choices. They are not prescriptive guidelines, but adaptable suggestions, which should be tailored to each individual. They are to be considered as a first step of a process that will continue to evolve as additional stakeholders provide new insights and as new information becomes available. As individualized risk prediction advances, the path to better tailored treatment regimens will become clearer.

Every capable clinician who treats Parkinson's disease (PD) implicitly understands the essential role of personalized treatment. Medications that may be appropriate for one patient may not be for another, and certain combinations may be particularly useful for one, but poorly tolerated by another. Clinical skills in this “art of medicine” are often unquantifiable, but are among the essential ingredients of excellence in clinical care.

There has been an expanding body of research focused on identifying clinical subtypes and predictors of PD outcomes, with varying success. Successful clinical subtyping has been limited by difficulties assigning patients to subtypes, migration of patients between subtypes, and variable or unknown power of subtypes to predict future events.^1^ Furthermore, some subtyping solutions can clearly distinguish different trajectories between groups of patients. Moreover, even beyond formal subtyping, there are established predictors of critical patient outcomes, including dementia, ability to walk independently, ability to live independently, etc.

Currently, it remains unclear to what degree advances in outcome prediction have translated into personalized medicine. Many clinicians have a broad sense of the likely outcomes of their patients. Many clinicians are also excellent at personalizing symptomatic treatment to the current state of their patients (eg, avoiding medications with potential cognitive side effects in those with cognitive impairment, selecting a single medication that may benefit multiple ongoing symptoms, etc.). Future considerations are incorporated into some treatment decisions for motor PD, for example using lower doses of dopaminergic medications or levodopa sparing strategies in young PD patients. Nevertheless, beyond the example of motor complications, clinicians currently possess limited tools to factor in the likelihood of future symptoms when making treatment decisions in the present. This may represent an important unmet need, as incorporating these future outcomes into current treatment decisions can yield important benefits. For example, if visits are infrequent, patients may suffer for months before a clinician recognizes a new opportunity to treat, or the need to remove a treatment that is now causing severe side effects. Many clinical manifestations have insidious onset and are, therefore, especially under‐recognized by patients and caregivers. Examples include cognitive decline, which requires intact cognition to recognize or psychiatric difficulties, which can be perceived as a new “normal” consequence of physical limitations. Additionally, some conditions are intermittent but serious (eg, falls because of syncope), with potential irreversible consequences. Therefore, identifying those at higher risk of symptoms can help prevent adverse impacts on patients' quality of life.

To date, published recommendations for applying current knowledge of outcome prediction to current treatment decisions are lacking. The International Parkinson and Movement Disorders Society (MDS) Task Force on PD subtypes has been conducting a systematic review of the subtyping literature and developing position statements on the nature and role of PD subtypes and future directions of subtyping research in PD.^1^ As an extension of this work, we conducted a consensus process to suggest potential practical symptomatic treatment implications of clinical subtyping and outcome prediction.

## Methods

The primary method used was a modified Delphi approach. During an initial in‐person meeting, ideas were brainstormed, followed by a three subsequent iterations during which revisions and additional ideas were solicited by email. The central question for the panel was:

“Using what you know about genetic/biological/clinical subtypes (or any individual‐level predictors of outcome), what advice would you give for selecting symptomatic treatments for an individual patient now, based on what their subtype or individual characteristics predict about their future disease course?”

Additional parameters were set, namely:

(1) Recommendations were not to be construed as rules or specific guidelines that must be followed, but rather, as general consideration or advice, subject to constant re‐evaluation for each individual patient. The paradigm was that of a general good clinical practice “clinical pearl” or tip that one might pass along to a trainee or colleague.
(2) It was understood that most recommendations would not be based on controlled trial level of evidence. Rather, they would be based primarily on evidence from studies of outcome prediction and subtyping, combined with clinical experience and the logical implications of outcome prediction.
(3) The scope of the recommendations was limited to symptomatic therapy and its symptomatic effects. Disease modification/neuroprotection considerations were considered outside the scope of this exercise.
(4) The future component was an essential feature of the proposal that set it apart from other treatment recommendations. Therefore, the goal was not to make recommendations based on current state alone, for example, avoiding medications with anticholinergic properties in patients who already had cognitive impairment. Rather, the recommendations would be to avoid such agents in those at risk of cognitive impairment, based on their subtype. Conversely, those at low risk could potentially use these medications and capitalize on their unique benefits.

### Modified Delphi Process

After collection of all suggested potential recommendations, results were compiled and edited for clarity/overlap (by R.P.). Each recommendation was sent to all members of the MDS task force on subtypes in PD. A total of 13 of the task force members had clinical expertise and participated in the modified Delphi process. Members who were non‐clinicians (ie, no direct experience of treating PD) did not participate in the panel.

For each suggestion, respondents answered:

“Do you agree that this is a generally‐useful clinical tip, and should be included?”

There were four possible responses to the question (with additional feedback and comments encouraged, which was then shared anonymously with the group).

(1) Yes (comments encouraged but not mandatory).
(2) Yes, but with certain suggestions (provide suggestion).
(3) Maybe yes, but essential modification is needed (provide specific modification below).
(4) No (provide reason below).

Following the first round of feedback, responses with fewer than 67% yes/maybe yes responses (ie, responses 1–3, combined) were eliminated. Based on feedback, each item was then revised for clarity, comprehensiveness, inclusion, etc. The revised wording was then submitted to the group for endorsement. Items with >75% endorsement were included in this manuscript. Items not retained after consensus are listed in the Supporting information section.

## Results

**Fig. 1:**
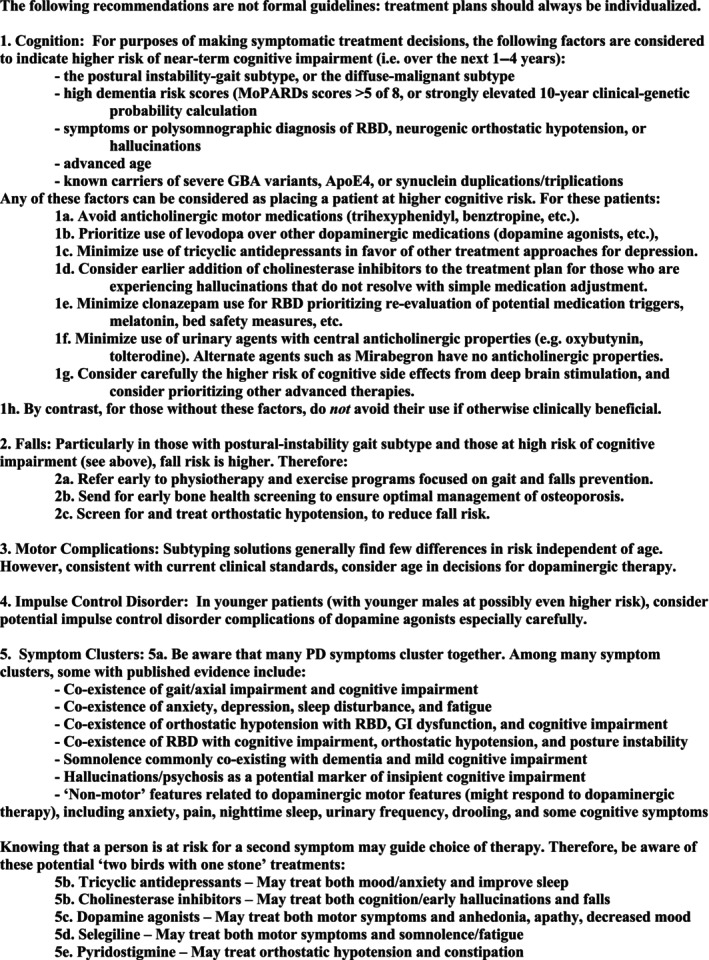
Task force recommendations for incorporation of future outcomes into symptomatic therapy.

Results are summarized in Figure 1.

1. Cognitive impairment

The largest number of recommendations concerned cognitive impairment. Cognitive changes can be both under‐recognized and have a significant functional impact. Even with adequate surveillance, cognitive deficits can be missed, or may emerge dramatically between infrequent visits (particularly if additional illnesses trigger acute decline). Many medications used for PD have potential cognitive side effects. However, these same medications are among the most useful for controlling PD symptoms, so blanket advice to avoid them can result in missed opportunity to improve quality of life.

Although it is impossible to fully quantify the risk, there is increasing evidence that cognitive impairment and dementia are associated with certain risk factors in PD, including:

(1) Advanced age (continuously increasing risk, with no clear cutoff)^2^, ^3^, ^4^, ^5^, ^6^
(2) Sleep disorders–particularly rapid eye movement (REM) sleep behavior disorder (RBD)^4^, ^7^, ^8^, ^9^, ^10^, ^11^
(3) Neurogenic orthostatic hypotension^2^, ^3^, ^12^, ^13^
(4) Other autonomic symptoms, in particular constipation^13^, ^14^, ^15^, ^16^
(5) Male sex^3^, ^4^, ^6^, ^17^, ^18^
(6) Prominent gait disorder (currently experiencing falls or freezing), and advanced motor disease^2^, ^4^, ^6^, ^19^, ^20^
(7) Mutations in *GBA*, *ApoE4*, and synuclein duplications/triplications (noting that this information may be unavailable in most clinical settings)^6^, ^7^, ^18^, ^21^, ^22^, ^23^
(8) Vascular risk factors or documentation of cerebrovascular disease^4^, ^17^
(9) Low level of education^6^, ^17^, ^24^, ^25^
(10) Early symptoms—hallucinations (including any hallucination consistent with PD pathology, and excluding those entirely because of alternate causes such as visual disturbance, etc.), mild cognitive changes^2^, ^4^, ^26^, ^27^

Although many of these single factors are well established, it is unclear to what degree they are powerful enough to change treatment decisions. To be clinically relevant, a risk factor would generally require a substantial increased risk (to justify modifying individual treatment decisions based only on future side effects). According to most studies, the risk factors of male sex, low education and vascular risk may fall below this threshold (eg, relative risk <2).

Additionally, increased dementia risk has been observed in certain subtypes of PD. However, most subtyping solutions do not include a method to classify individual patients into a subtype, and therefore, cannot be applied in clinical practice. Two subtyping solutions that do allow for individual classification include postural‐instability gait disturbance (PIGD) subtypes (which are generally defined according to Unified Parkinson's Disease Rating Scale [UPDRS] or MDS‐UPDRS),^2^, ^24^, ^28^ and the diffuse‐malignant subtype of PD (which is defined by assessing MDS‐UPDRS, orthostatic hypotension, REM sleep behavior disorder, and cognitive impairment).^19^, ^29^, ^30^

Finally, two dementia risk predictors have been developed for use in the office setting. A clinical‐genetic score, developed from a multicenter cohort, translates clinical‐genetic variables (age, sex, years of education, the Mini‐Mental State Examination score, MDS‐UPDRS part III, depression, and glucocerebrosidase (GBA) status) into a 10‐year dementia risk, with an on‐line calculator included.^6^ The long timescale of 10 years makes it difficult to translate risk scores into practical treatment considerations for the near future, and this precluded our selection of a specific cutoff. Regardless, a highly elevated 10‐year risk would likely also translate into a moderate‐to‐high risk over the shorter term. A second office‐based tool, the Montreal Parkinson Risk of Dementia Scale (MoPARDS) combines eight clinical variables to predict dementia: age >70, male sex, falls or freezing, bilateral disease onset, symptoms of RBD, orthostatic hypotension, hallucinations, and mild cognitive impairment, which are simply added to generate a risk score.^5^ The annual conversion rate to dementia among individuals with >5 factors was 14.9%, compared with 5.8% among those with 4 or 5 factors, and 0.6% among those with 0–3 factors.^3^, ^5^ Therefore, the following recommendation was adopted.

For purposes of making symptomatic treatment decisions, the following factors are considered to indicate higher risk of near‐term cognitive impairment (ie, over the next 1–4 years):

(1) The postural instability‐gait disturbance subtype (for patients who are known carriers of G2019S *LRRK‐2* mutations, consider that dementia risk is lower, even in those with significant postural‐instability and gait symptoms. Do not use PIGD subtyping alone to define cognitive risk in these patients.)^9^, ^20^, ^24^, ^28^, ^31^
(2) The diffuse‐malignant subtype^29^, ^30^, ^32^
(3) High dementia risk scores (MoPARDs scores >5 of 8^3^, ^5^ or strongly elevated 10‐year clinical‐genetic probability calculation^6^
(4) Symptoms or polysomnographic diagnosis of RBD
(5) Neurogenic orthostatic hypotension
(6) Hallucinations
(7) Advanced age
(8) Known carriers of severe GBA variants, ApoE4, or synuclein duplications/triplications

Any of these factors can be considered as placing a patient at higher cognitive risk.

Treatment recommendations based on risk of cognitive side effects.

The panel made the following suggestions with regards to those at high risk of near‐term cognitive impairment:

1a Avoid anticholinergic motor medications (trihexyphenidyl, benztropine, etc.)
1b Prioritize use of levodopa over other dopaminergic medications (dopamine agonists, etc.)
1c Minimize use of tricyclic antidepressants in favor of other treatment approaches for depression.
1d Consider earlier addition of cholinesterase inhibitors to the treatment plan for those who are experiencing hallucinations that do not resolve with simple medication adjustment. By contrast, for those with low dementia risk, response to cholinesterase inhibition may be less. Therefore, prioritize alternate treatments such as medication adjustment, prescription of an atypical antipsychotics, etc. (noting that these alternate treatments should also be considered in those with high cognitive risk).
1e Minimize clonazepam use for RBD, prioritizing re‐evaluation of potential medication triggers (eg, antidepressants), melatonin, bed safety measures, etc.
1f Minimize use of urinary agents with central anticholinergic properties (eg, oxybutynin, tolterodine). Alternate agents such as Mirabegron have no anticholinergic properties.
1g Consider carefully the higher risk of cognitive side effects from deep brain stimulation and consider prioritizing other advanced therapies.
1h By contrast, for those with a low risk of dementia (young age, absence of RBD/orthostatic hypotension/hallucinations, tremor‐dominance, pure motor subtypes, low dementia risk scores, etc.) carefully consider the potential benefits of all the above interventions (1a–1g) relative to the lower risk of cognitive side effects, and do not avoid their use if otherwise clinically beneficial.

2 Falls

Although the literature on the relationship between subtypes and falls is limited, it is evident that those with the PIGD subtype of PD are, by definition, at increased fall risk. In addition, a single study found that the diffuse‐malignant subtype of PD had a threefold shorter latency to falls than those with the pure‐motor subtype.^19^ Evidence is also accumulating that those with dementia have higher risk of falls, and there are substantial overlaps between risk factors for falls and dementia.^19^, ^33^, ^34^, ^35^ Most other single‐item predictors of falls center around objective gait measures or markers of advanced disease, in which case fall risk should be already evident. Orthostatic hypotension is another common and treatable cause of falls and is linked to both cognitive impairment and postural‐instability gait subtypes^30^ (therefore, all advice from item 1 applies to this group as well). Cholinesterase inhibitors may prevent falls even in those without cognitive impairment,^36^ although confirmatory trials are pending.^37^ The primary method for fall prevention is consultation with allied health, and the primary consequence of falls is injury, so the following recommendations were made.

2 Particularly in those with postural‐instability gait subtype and those at high risk of cognitive impairment (see Section 1), fall risk is higher. Therefore:

2a Refer early to physiotherapy and exercise programs focused on gait and fallprevention
2b Send for early bone health screening to ensure optimal management of osteoporosis because of higher risk of falls and fractures
2c Screen for and treat orthostatic hypotension, to reduce fall risk

3 Motor fluctuations

The emergence of motor fluctuations and dyskinesia is a primary concern in PD treatment. Many studies have been performed to identify risk factors for motor complications. Putative risk factors include younger age, longer disease duration, anxiety and depression, high MDS‐UPDRS part 1 scores, greater levodopa response, female sex (with and without factoring in body mass index), among others.^38^, ^39^, ^40^ Among these factors, age has emerged as the consistent across studies, with younger patients generally displaying better levodopa response but experiencing more fluctuations and dyskinesia. As a result, levodopa is usually suggested as first line therapy in those who are older (eg, age >70) whereas for those who are younger, choice of first line therapy varies according to the degree of disability, co‐morbidities, individual patient preferences, etc. Interestingly, most subtyping approaches with age‐matched groups did not find substantial differences in fluctuations or dyskinesia between subtypes. Noting that this aspect is less novel than the panel's other recommendations, the well‐established relationship between age and motor complications is underscored in the following recommendation:

3 In general, subtyping solutions have not found differences in risk of dyskinesia or fluctuations independent of age. However, consistent with current clinical standards, consider age in decisions for dopaminergic therapy.
4 Impulse control disorders

Impulse control disorders (ICDs) are among the most feared complications associated with dopaminergic treatment, particularly with dopamine agonists. ICDs often remain subclinical or are actively concealed by patients, yet their impact on quality of life can be severe. This makes them prime candidates for a risk‐based approach. Putative risk factors for ICD's include younger age, premorbid personality (risk taking behavior, novelty seeking, smoking, etc.), depression, and male sex.^41^, ^42^, ^43^ Premorbid personality is often not accurately measured in a clinical setting, and prior impulse control disorder is already an obvious contraindication to dopamine agonists. Notably, although depression is a risk factor for ICDs, it may also be potentially improved by dopamine agonists,^44^ questioning a blanket contraindication recommendation. In all cases, the risk of ICD's should be balanced with potential benefits, and the risk should be extensively discussed with the patient and the care partner. Given the strong relationship between age, with an additional likely contribution of sex, the following recommendation was made:

4 In younger patients (with younger males at possibly even higher risk), consider potential impulse control disorder complications of dopamine agonists especially carefully, as these subgroups are at higher risk of these complications.
5 Symptom clusters and “two birds with one stone” treatments.

Whereas subtyping studies attempt to classify patients into clusters, there are also clusters of symptoms in PD. Most have not been systematically studied in formal data analytic approaches.^45^, ^46^ Symptom clusters may be related to common shared mechanisms (eg, serotonergic systems, etc.), or to shared patterns of neurodegeneration. Being aware of these clusters can help clinicians to screen carefully for possible under‐recognized associated symptoms. Beyond this, it may even guide therapeutic choice; if several competing treatments options are available for a symptom, choosing the one that may also help another subclinical or latent symptom can further improve quality of life. The following recommendation was approved:

5a Be aware that many PD symptoms cluster together. Therefore, if one symptom is present, extra surveillance is required for the other symptoms that often accompany this. In some cases, this might even guide initial treatment of a specific symptom (ie, in the anticipation that other commonly linked symptoms may soon arrive). Among many symptom clusters, some with published evidence include:

- Co‐existence of gait/axial impairment and cognitive impairment^20^, ^28^, ^47^
- Co‐existence of anxiety, depression, sleep disturbance, and fatigue^45^, ^46^
- Co‐existence of orthostatic hypotension with RBD, gastrointestinal dysfunction, and cognitive impairment^2^, ^13^, ^29^, ^30^, ^48^, ^49^
- Co‐existence of RBD with cognitive impairment, orthostatic hypotension, and posture instability^8^, ^10^, ^11^, ^50^, ^51^, ^52^
- Co‐existence of somnolence with dementia and mild cognitive impairment^11^, ^53^
- Hallucinations/psychosis as a potential marker of incipient cognitive impairment^2^, ^4^, ^27^
- “Non‐motor” features, which are related to dopaminergic motor features (and therefore, might respond to dopaminergic therapy), including anxiety, pain, nighttime sleep, urinary frequency, drooling, and some cognitive symptoms.^54^, ^55^, ^56^, ^57^

Knowing that a person is at risk for a second symptom may guide choice of therapy.

Therefore, be aware of these potential “two birds with one stone” treatments:

5b Tricyclic antidepressants – May treat both mood/anxiety^58^ and improve sleep (randomized trials find reduction in insomnia and improved sleep efficacy)^59^, ^60^, ^61^, ^62^
5c Cholinesterase inhibitors – May treat both cognition/early hallucinations and falls^36^
5d Dopamine agonists – May treat both motor symptoms and anhedonia, apathy, ordecreased mood^44^
5e Selegiline – May treat both motor symptoms and somnolence/fatigue (selegiline is metabolized to amphetamines)^63^, ^64^, ^65^
5f Pyridostigmine – May treat orthostatic hypotension and constipation (evidence for pyridostigmine's efficacy remains limited)^66^, ^67^, ^68^

## Discussion

Research on PD subtypes and outcome predictors in general holds significant potential for various applications. Here, we focused on a specific immediate clinical implication: using the insights gained from subtyping and outcome prediction research to offer practical treatment recommendations for healthcare providers and patients living with PD. The core idea proposed is to better integrate anticipated future outcomes into current treatment decisions. The recommendations centered primarily around two key insights, namely (1) to formally incorporate risk of future cognitive impairment into treatment decisions; and (2) to recognize common symptom clusters and opt for treatments that might achieve a dual purpose (ie, “two birds with one stone” approach).

It is important to emphasize that these recommendations represent only a beginning. The list of recommendations is by no means comprehensive; many more can be suggested in the future. The current recommendations were limited to clinical symptomatic treatments using clinical predictors of outcome. Markers of biological subtypes or indicators of biological heterogeneity were not included, because their implications for clinical symptomatic treatment decisions remain unclear. The current recommendations reflect the consensus of a single group of researchers in the field of PD subtypes and should not be considered an official stance by the MDS or any other organization. Because of the scope of our question, recommendations generally would not have randomized controlled trial support. Rather they were generated from the logical implications of outcome prediction studies, combined with clinical experience. As such, these recommendations should be considered as complementary to those coming from rigorous evidence‐based/grading of recommendations assessment, development, and evaluation methodology. Finally, and most critically, these are not to be interpreted as guidelines that should be invariably followed. Rather these should be interpreted as general “rules of thumb” that can help make individual treatment decisions, always in the context of individual patient preferences, comorbidities, and other factors, and by a clinician with expertise in PD. These individual circumstances will differ considerably, necessitating the development of a personalized treatment plan. The goal of these recommendations is to introduce a method of incorporating outcome prediction knowledge into current clinical care in ways that may not be immediately apparent to clinicians.

As we look to the future, what will improve our ability to plan treatment based on future outcomes? A critical need is to continue to expand the scope and analysis of large patient cohorts. In particular, long follow‐up duration is essential to capture the full range of outcomes, including those that occur predominantly in later disease and those that emerge with the chronic use of certain medications. To be clinically useful, models must not only predict overall prognosis, but rather be able to predict individual disease manifestations. In this regard, the office‐based dementia risk calculators (clinical‐genetic risk calculator and the MoPARDS)^5^, ^6^ are potential models. To date, neither appear to have been adopted into general use, underscoring the need for further dissemination. As knowledge deepens, individual prediction algorithms can be refined to provide risks of each patient milestone at any specific time interval, allowing increasingly precise refinement of individual treatment plans.

## Author Roles

(1) Research project: A. Conception, B. Organization, C. Execution; (2) Manuscript Preparation: A. Writing of the First Draft, B. Review and Critique.

R.P.: 1A, 1B, 1C, 2A.

D.W., T.S., M.R.V., A.L., M.H., A.E., R.E., K.D., N.B., D.B., T.M., C.M.: 1C, 2B.

## Disclosures

**Ethical Compliance Statement:** We confirm that we have read the Journal's position on issues involved in ethical publication and affirm that this work is consistent with those guidelines. The authors confirm that the approval of an institutional review board was not required for this work. The authors confirm that patient consent was not required for this work.

**Funding Sources and Conflict of Interest:** No specific funding was received for this work. The authors declare that there are no conflicts of interest relevant to this work.

**Financial Disclosures for the Previous 12 Months:** R.B.P. reports grants and personal fees from Fonds de la Recherche en Sante, grants from Canadian Institute of Health Research, The Michael J. Fox Foundation (MJFF), the Webster Foundation, Roche, and the National Institute of Health and personal fees from Takeda, Biogen, AbbVie, Curasen, Lilly, Novartis, Eisai, Paladin, Merck, Vaxxinity, Korro, Bristol Myers Squibb and the International Parkinson and Movement Disorders Society, outside the submitted work. D.W. has received research funding or support from The Michael J. Fox Foundation for Parkinson's Research, Alzheimer's Therapeutic Research Initiative, Alzheimer's Disease Cooperative Study, International Parkinson and Movement Disorder Society, National Institute on Health (NIH), The Parkinson's Foundation and the United States Department of Veterans Affairs; honoraria for consultancy from Alkahest, Aptinyx, Boehringer Ingelheim, Cerevel Therapeutics, CHDI Foundation, CuraSen, Ferring, Medscape, Roche, Sage, Scion, Signant Health and Takeda; and license fee payments from the University of Pennsylvania for the QUIP and QUIP‐RS. T.S. has served as a consultant for AcureX, Adamas, AskBio, Amneal, Blue Rock Therapeutics, Critical Path for Parkinson's Consortium, Denali, MJFF, Neuroderm, Sanofi, Sinopia, Roche, Takeda and Vanqua Bio. T.S. served on the ad board for AcureX, Adamas, AskBio, Denali, and Roche. T.S. has served as a member of the scientific advisory board of Koneksa, Neuroderm, Sanofi, and UCB. T.S. has received research funding from Amneal, Biogen, Neuroderm, Prevail, Roche, and UCB, and an investigator for National Institute of Neurological Disorders and Stroke, MJFF, Parkinson's Foundation. M.R.V. has received honoraria from Boston Scientific, Ever Neuropharma, Abbot, Ferrer, Silanes, and Dumont; and stipends from the International Parkinson and Movement Disorders Society and World Parkinson Coalition, outside the submitted work. A.F.G.L. receives royalties from De Tijdstroom publishers and Springer media. M.H. reports grant support from Parkinson's United Kingdom, Oxford National Institute for Health and Care Research (NIHR) Biomedical Research Centre, University of Oxford, CPT, Lab10X, NIHR, MJFF, H2020 European Union, GE Healthcare and the PSP Association. She also received payment for Advisory Board. A.J.E. has received grant support from the NIH and MJFF; personal compensation as a consultant/scientific advisory board member for Neuroderm, Amneal, Acadia, Avion Pharmaceuticals, Acorda, Kyowa Kirin, Supernus (formerly, US WorldMeds), and Herantis Pharma; personal honoraria for speakership for Avion, Amneal, and Supernus; and publishing royalties from Lippincott Williams and Wilkins, Cambridge University Press, and Springer. He cofounded REGAIN Therapeutics and is co‐inventor of the patent “Compositions and methods for treatment and/or prophylaxis of proteinopathies.” He serves on the editorial boards of the Journal of Parkinson's Disease, Journal of Alzheimer's Disease, European Journal of Neurology, Movement Disorders Clinical Practice, and JAMA Neurology. R.E. has received grants from the Italian Ministry of Health and MSA Coalition, personal fees from Ipsen and the International Parkinson and Movement Disorders Society and royalties from Springer, outside the submitted work. K.D. has no conflict of interest to declare. N.I.B. has received grant support from the NIH, Department of Veterans Affairs, Parkinson's foundation, and Farmer Family Foundation. He has received in kind research support from SynOne, Neurolign USA, Scion Neurostim. He is the CEO of Tulip M3D. He is part of an outside start‐up called Tulip Tables Make Me Move, which does not provide financial or non‐financial support for this project; does not supply a product; does not perform work on this project but holds a possible option to license investigator/UM IP in the future. D.B. received consultancies/advisory board fees of ACImmune, Lilly, UCB Pharma; honoraria for talks/lectures from ACImmune, Biogen, UCB Pharma, Novartis grants/research funding from Deutsche Forschungsgemeinschaft (DFG), German Parkinson's Disease Association (dPV), BMBF, Parkinson Fonds Deutschland, UCB Pharma, EU, Novartis Pharma, Lundbeck, and the Damp Foundation. T.A.M. reports speaker honorarium from AbbVie; consultancies from CHDI Foundation/Management, Roche; advisory board from AbbVie; Biogen, and research funding from EU Joint Programme‐Neurodegenerative Disease Research, uOBMRI, Roche, Ontario Research Fund, Canadian Institutes of Health Research, MJFF, Parkinson Canada, PDF/PSG, LesLois Foundation, PSI Foundation, Parkinson Research Consortium. C.M. is a site PI for the Parkinson's Progression Markers Initiative study and receives funding from MJFF for this role.

## Supporting information


**Data S1.** Summary of suggested recommendations that were not endorsed after consensus.
