# Supplementary material for: Anticipating Tomorrow: Tailoring Parkinson's Symptomatic Therapy Using Predictors of Outcome
Source: Mov Disord Clin Pract. 2024 May 30;11(8):983–91. doi: 10.1002/mdc3.14089 (PMC11329576; doi:10.1002/mdc3.14089)
Supplement: Supplementary file 1 — Data S1. Summary of suggested recommendations that were not endorsed after consensus. [file MDC3-11-983-s001.docx]

Supplementary section

Items removed from Delphi consensus

The following items were suggested by panelists, but did not meet threshold for inclusion. Note that those suggestions that were ineligible because out of the scope of the panel (e.g. suggestions for potential neuroprotection, suggestions in which outcome prediction was not relevant, etc.) were pre-screened and not voted upon by the panel, and so are not included in this list. Rewording edits, minor clarifications, etc., are not included in this list.

1. **For purposes of making symptomatic treatment decisions, the following factors are considered to indicate higher risk of near-term cognitive impairment (i.e., over the next 1-4 years):**

Removed items, with rationale:

- *vascular risk factors* – insufficient prognostic strength of effect to alter symptomatic treatment

- *low education* - insufficient prognostic strength of effect to alter symptomatic treatment

- *male sex* - insufficient prognostic strength of effect to alter symptomatic treatment

- *depression* – insufficient prognostic strength of effect to alter symptomatic treatment

- *those known to have co-morbid AD pathology* –not broadly applicable, as documentation of AD pathology in most clinical setting (e.g. PET scan) is very uncommon

- *diabetes* – insufficient prognostic strength of effect to alter symptomatic treatment

- *synuclein mutations* – too rare to warrant a broad inclusion

- *apathy* – insufficient evidence for effect

1. In section of specific recommendations

**1a. . . .** *avoid anticholinergics and limit dose of agents with potential anticholinergic properties (e.g. Amantadine) –* Amantadine removed from specific mention because evidence for clinically-significant anticholinergic effects at lower doses was thought to be insufficient for broad recommendation. Specific anticholinergic motor medications (trihexyphenidyl, benztropine) were listed instead.

*4b. For patients who are carriers of G2019S LRRK-2 mutations, have a high index of suspicion for anxiety and depression, and consider use of tricyclic antidepressants, SSRI medications, dopamine agonists, etc., when appropriate*

- Rejected for insufficient consensus – one would wait for depression before treating (i.e., no strong anticipatory component), limited evidence, insufficient diagnostic strength (depression/anxiety common in all PD)

6. **Therefore, be aware of these potential ‘two birds with one stone’ treatments:**

- *Myrbetriq for bladder dysfunction and orthostatic hypotension* – effect of Myrbetriq upon blood pressure in PD was warranted too modest / equivocal to warrant recommendation

- Tricyclic antidepressants – *possible mild antiparkinsonian effect (anticholinergic) –* removed for insufficient evidence – other benefits (insomnia and depression) retained in text

- *Amantadine for dyskinesia and impulse control disorders* – potential impulse control disorder benefit considered to have insufficient evidence for broad recommendation

- *Rivastigmine for cognition and orthostatic hypotension* – potential orthostatic hypotension benefit considered to have insufficient evidence for broad recommendation

*- Methylphenidate for excessive daytime sleepiness and cognitive impairment and OH –* potential orthostatic hypotension and cognitive benefit considered to have insufficient evidence for broad recommendation
